# Discovery and characterization of natural tropolones as inhibitors of the antibacterial target CapF from *Staphylococcus aureus*

**DOI:** 10.1038/srep15337

**Published:** 2015-10-16

**Authors:** Koichiro Nakano, Takeru Chigira, Takamitsu Miyafusa, Satoru Nagatoishi, Jose M. M. Caaveiro, Kouhei Tsumoto

**Affiliations:** 1 Department of Medical Genome Sciences, Graduate School of Frontier Sciences, The University of Tokyo, 4-6-1 Shirokanedai, Minato-ku, Tokyo 108-8639, Japan; 2 Department of Chemistry and Biotechnology, School of Engineering, The University of Tokyo, Bunkyo-ku, Tokyo 113-8656, Japan; 3 Institute of Medical Science, The University of Tokyo, 4-6-1, Shirokanedai, Minato-ku, Tokyo 108-8639, Japan; 4 Biomedical Research Institute, National Institute of Advanced Industrial Science and Technology, Central 6, 1-1-1 Higashi, Tsukuba, Ibaraki 305-8566, Japan; 5 Department of Bioengineering, School of Engineering, The University of Tokyo, 7-3-1 Hongo, Bunkyo-ku, Tokyo 113-8656, Japan

## Abstract

The rapid spread of antibiotic-resistance among pathogenic bacteria poses a serious risk for public health. The search for novel therapeutic strategies and antimicrobial compounds is needed to ameliorate this menace. The bifunctional metalloenzyme CapF is an antibacterial target produced by certain pathogenic bacteria essential in the biosynthetic route of capsular polysaccharide, a mucous layer on the surface of bacterium that facilitates immune evasion and infection. We report the first inhibitor of CapF from *Staphylococcus aureus*, which was identified by employing fragment-based methodologies. The hit compound 3-isopropenyl-tropolone inhibits the first reaction catalyzed by CapF, disrupting the synthesis of a key precursor of capsular polysaccharide. Isothermal titration calorimetry demonstrates that 3-isopropenyl-tropolone binds tightly (*K*_*D*_ = 27 ± 7 μM) to the cupin domain of CapF. In addition, the crystal structure of the enzyme-inhibitor complex shows that the compound engages the essential Zn^2+^ ion necessary for the first reaction catalyzed by the enzyme, explaining its inhibitory effect. Moreover, the tropolone compound alters the coordination sphere of the metal, leading to the overall destabilization of the enzyme. We propose 3-isopropenyl-tropolone as a precursor to develop stronger inhibitors for this family of enzymes to impair the synthesis of capsular polysaccharide in *Staphylococcus aureus*.

*Staphylococcus aureus* (*S. aureus*) is a Gram-positive opportunistic bacterium causing life-threatening infections such as sepsis, endocarditis and pneumonia^1^,^2^. Alarmingly, the treatment against *S. aureus* is increasingly ineffective owing to the emergence of methicillin- and vancomycin-resistant strains^3^,^4^. The proliferation of antibiotic-resistant strains of *S. aureus* thus represents a therapeutic challenge and a menace for public health. For these reasons, efforts to find novel targets to overcome antibiotic resistance are in urgent need^5^.

The capsular polysaccharide (CP) is a polymeric carbohydrate attached to the outer surface of *S. aureus* critical for bacterial resistance because of its role in reducing the immune response of the host^6^,^7^. Targeting the synthesis of CP is therefore a potential therapeutic avenue to combat pathogenic strains of *S. aureus*. Most clinical isolates of *S. aureus* express either serotype 5 or serotype 8 CP composed of repeating units of L-FucNAc, D-FucNAc, and D-ManA. In particular, the biosynthetic pathway of the CP precursor UDP-L-FucNAc is well conserved among several pathogenic bacteria^8^. Targeting enzymes belonging to this pathway with inhibitors opens innovative strategies for the discovery of new therapeutic agents.

In *S. aureus*, the enzymes CapE, CapF and CapG catalyze the sequential transformation of UDP-D-GlcNAc in the CP precursor UDP-L-FucNAc *via* the intermediate compound UDP-N-acetyl-L-talosamine (UDP-L-TalNAc) (Fig. 1)^9^,^10^. Importantly, knockout and complementation studies have demonstrated the essential role of these enzymes for the synthesis of serotype 5 CP, justifying their potential as antibacterial targets^8^.

In previous studies we and others characterized the bifunctional enzyme CapF from structural, thermodynamic, and biochemical standpoints, laying the groundwork for the identification and characterization of inhibitors and drug-like compounds^9^,^10^,^11^,^12^. Structurally, CapF is a homo-dimer displaying a characteristic dumb-bell shaped architecture composed of two domains, each one catalyzing separate enzymatic reactions (Fig. 1)^12^. The C-terminal cupin domain of CapF catalyzes the epimerization of the compound produced by the upstream enzyme CapE, whereas the N-terminal SDR domain catalyzes the reduction of the compound afforded by the cupin domain, requiring one equivalent of NADPH. CapF is a metalloenzyme containing a Zn^2+^ ion in the cupin domain crucial for catalyzing the first chemical reaction, and also important for the stability of the enzyme^12^.

Here we have performed a screen with a small-molecule chemical library of fragments to identify compounds binding to and inhibiting the enzymatic activity of CapF. We describe the inhibition of the first reaction catalyzed by CapF by natural tropolones (7-membered ring aromatic compounds).Calorimetry and X-ray crystallography demonstrate that 3-isopropenyl-tropolone binds to the pocket of the cupin domain of CapF. The hit compound chelates the critical Zn^2+^ ion and exhibits specific non-covalent interactions with the enzyme as evidenced by the favorable binding enthalpy and its coordination chemistry. We discuss potential routes to increase the potency of this novel inhibitor of CapF.

## Results

### Identification of a novel inhibitor of CapF

To identify small-molecule inhibitors of CapF we employed fragment-based methodologies (Supplementary Information Figure S1)^13^,^14^. A fragment library consisting of 1,994 compounds was obtained from the Drug Discovery Initiative program (The University of Tokyo, see the Methods section for an brief description of the features of this library). This library was employed to screen for potential inhibitors of CapF using the technique of surface plasmon resonance (SPR). This screening methodology identified compounds with the ability to bind to the target protein but not by their inhibitory potency. Inhibition was only assessed with the selected hit compound using two separate enzymatic assays (see below). To maximize the sensitivity of the SPR signal the microtiter-based assay mode was employed. The values of the parameters CV (1.0%) and Z-factor (0.93) were monitored throughout the screen^15^. Fragments displaying box-type kinetic responses within the top 10% binding responses were considered for further characterization (Supplementary Information Figure S1)^16^,^17^.

**Figure 2:**
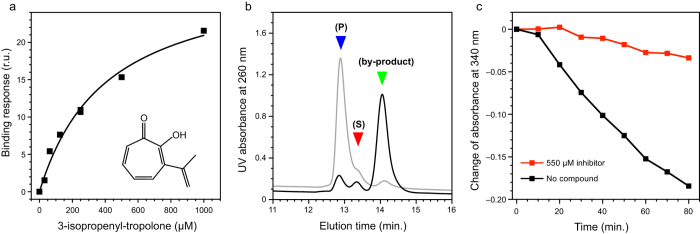
Validation of a novel inhibitor of CapF. (**a**) Binding response of 3-isopropenyl-tropolone to a surface decorated with CapF. The structural formula of 3-isopropenyl-tropolone is shown. (**b**) Enzymatic assay in the absence (gray) and presence (black) of 1 mM inhibitor. Generation of product (indicated by the blue triangle) is inhibited in the presence of 3-isopropenyl-tropolone. Inhibition of CapF is conducive to the accumulation of the thermodynamically favored byproduct (green triangle) generated by the preceding enzyme CapE. (**c**) NADPH consumption by the SDR domain of CapF in the absence (black line and squares) or presence (red line and squares) of 3-isopropenyl-tropolone.

A total of 15 compounds selected from the library screening were next evaluated with SPR at three different concentrations (100 μM, 10 μM, and 1 μM) to detect compounds giving false positive signals, which are generally known as nonspecific and promiscuous binders. Five fragments exhibiting binding responses in a concentration-dependent manner were selected for additional evaluation. Lastly, we determined the binding affinity of these five compounds for CapF with SPR. Only 3-isopropenyl-tropolone bound to CapF with sub-millimolar affinity, a threshold we judged necessary to justify further analysis (Fig. 2a, and Supplementary Information Figure S1e). The other four compounds displayed low affinities (mM range) and were not considered for additional examination.

Although 3-isopropenyl-tropolone binds robustly to CapF, as shown above, it was necessary to verify whether it also inhibits the enzymatic activity of the protein or not. For that purpose, an HPLC-based assay monitoring the consumption of substrate and the appearance of product was employed (Fig. 2b)^12^. Whereas in the absence of 3-isopropenyl-tropolone the substrate was converted into product with nearly quantitative yield (91%), in the presence of 1 mM 3-isopropenyl-tropolone the peaks corresponding to substrate and product were largely absent (their combined areas were <18%). Instead, a large peak corresponding to the byproduct generated by the enzyme CapE under thermodynamic control was observed (80% yield as judged from the area under the curve). To confirm that inhibition of 3-isopropenyl-tropolone is specific for CapF we performed an additional experiment employing only CapE, which is the enzyme generating the substrate for CapF in the functional assay (Supplementary Information Figure S2). The HPLC profile of the reaction products was not altered in the presence of inhibitor, ruling out the possibility that the hit compound also inhibits CapE.

A second assay monitoring the consumption of NADPH corroborates the inhibitory effect of 3-isopropenyl-tropolone (Fig. 2c). This assay is sensitive to the NADPH consumed by the SDR domain during the second reaction catalyzed by CapF (reduction step). It was observed that the rate of consumption of NADPH slowed down as the concentration of inhibitor increases, indicating direct inhibition of CapF. The accumulation of the byproduct generated by CapE in the presence of inhibitor (Fig. 2b) suggested that inhibition occurs in the cupin domain of CapF. Nevertheless, additional experiments were required to corroborate that idea (see below).

### Zn^2+^ is required for the binding of inhibitor to CapF

**Figure 3:**
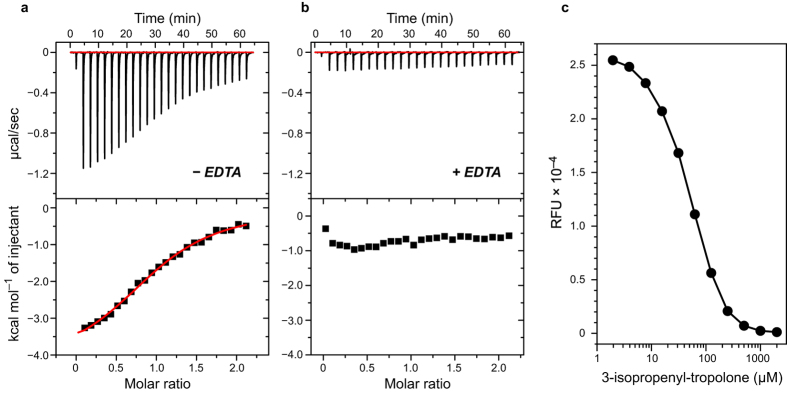
Binding of 3-isopropenyl-tropolone to CapF requires Zn^2+^. CapF (150 μM) reconstituted with Zn^2+^ was titrated with 1.65 mM inhibitor in the absence (**a**) or the presence (**b**) of 1 mM EDTA. The upper panels correspond to the titration data, and the lower panels to the integrated binding isotherms. (**c**) Binding competition assay. CapF (1.0 μM) reconstituted with Zn^2+^ in the presence of the Zn^2+^-sensitive dye FluoZin-3 (1.0 μM) was titrated with 3-isopropenyl-tropolone (λ_EX_ = 490 nm; λ_EM_ = 520 nm).

Because tropolone and its analogs have been described as metal chelators in proteins^18^, we anticipated that binding of the inhibitor to CapF is governed by the Zn^2+^ ion present in the cupin domain^12^. To confirm this hypothesis, we performed two different types of experiments. First, we determined the binding properties of 3-isopropenyl-tropolone to apo-CapF (*i.e*. Zn^2+^-depleted CapF) and to holo-CapF (reconstituted with Zn^2+^) by isothermal titration calorimetry (ITC). When holo-CapF was employed, the compound bound robustly as demonstrated by the exothermic sigmoid isotherm shown in Fig. 3a. The stoichiometry is consistent with a 1:1 binding model. The dissociation constant (*K*_*D*_) determined by non-linear regression was 27 ± 12 μM. In contrast, 3-isopropenyl-tropolone does not bind to CapF when apo-CapF (Zn^2+^-depleted) was employed (Fig. 3b). We note that binding of 3-isopropenyl-tropolone to free-Zn^2+^ in solution was not detected by ITC under our experimental conditions (Supplementary Information Figure S3a), suggesting that specific interactions between Zn^2+^ ion and the inhibitor occur only in the context of the binding pocket of the enzyme.

Second, the fluorescence of the Zn^2+^-specific indicator FluoZin-3^19^,^20^ was examined in the presence of CapF. The signal of FluoZin-3 increases dramatically in the presence of Zn^2+^ ions when the metal is accessible to the dye. Hence, the high fluorescence signal of the indicator in the presence of holo-CapF indicated that Zn^2+^ is exposed in the pocket of the cupin domain for interaction with the fluorescent dye (Fig. 3c, and Supplementary Information Figure S3b). This result is consistent with the location of Zn^2+^ in a region accessible to the solvent, as previously reported in the crystal structure of CapF^12^. In contrast, the fluorescence of FluoZin-3 with apo-CapF, or with holo-CapF in the presence of 3-isopropenyl-tropolone, was negligible. We also observed that, in the presence of holo-CapF, the fluorescence signal of FluoZin-3 depends on the concentration of hit compound in solution, exhibiting a sigmoid shape with an inflexion point at 51 μM (Fig. 3c).

A control experiment with CapF lacking the His_6_-tag was carried to rule out the possibility of spurious effects caused by the affinity tag (Supplementary Information Figure S4). The titration profile and thermodynamic parameters corresponding to the binding of 3-isopropyl-tropolone to CapF lacking the His_6_-tag are very similar to those observed with the tagged enzyme, thus demonstrating the affinity-tag bears negligible influence for the interaction between enzyme and inhibitor. Collectively, the data above clearly suggest that the key Zn^2+^ ion in the cupin domain is essential for the binding of 3-isopropenyl-tropolone to the enzyme.

### Crystal structure of the inhibitor bound to CapF

To examine the structural basis for the binding of 3-isopropenyl-tropolone to CapF we employed X-ray crystallography. A construct lacking the loop 57-70 at the N-terminal SDR domain (CapFΔ57-70) was employed for the structural characterization of the enzyme-inhibitor complex. Although this loop does not interact with the cupin domain in the structure of the dimer, it establishes intermolecular interactions with another dimer of CapF in the crystal form (PDB entry codes 3ST7 and 3VHR)^12^. In these two independent crystal structures of WT CapF, it is observed that residues belonging to the loop 57-70 of one dimer of CapF insert in the Zn^2+^ pocket of the cupin domain of a different dimer, presumably stabilizing the crystal lattice (Supplementary Information Figure S5). This crystallographic artifact may interfere with binding of 3-isopropenyl-tropolone to the Zn^2+^-pocket in soaking experiments with crystals of WT-CapF by blocking access of the compound. To avoid this severe complication, we employed the truncated construct CapFΔ57-70 and successfully obtained the structure of the enzyme-inhibitor complex. These intermolecular interactions are negligible in solution, since CapF is dimer, and not a tetramer or higher assembly^12^. We also note that the truncation does not modify the thermal stability of the resulting enzyme with respect to WT CapF as judged from the values of the mid-point transition (*T_M_*) obtained by differential scanning calorimetry (DSC) (*ΔT*_*M*_ < 1 °C, Table 1).

**Figure 4:**
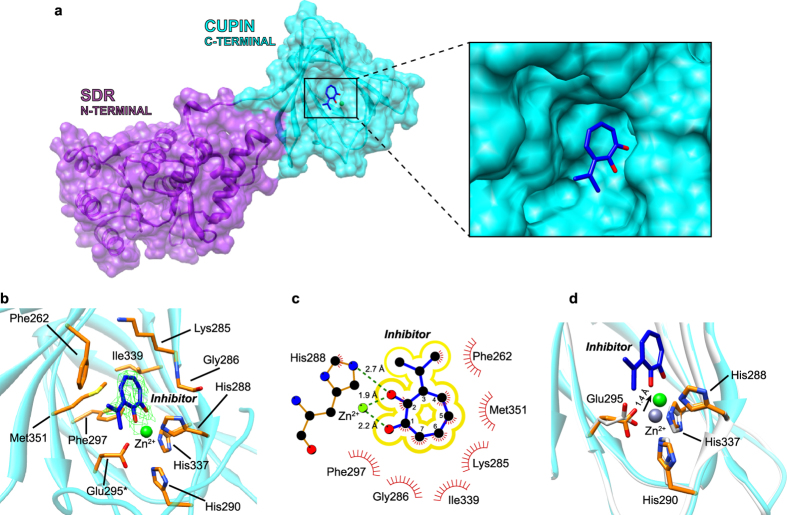
Crystal structure of CapFΔ57-70 in complex with 3-isopropenyl-tropolone. (**a**) Overall structure of one protein chain. The compound (blue sticks) binds in the active site of the cupin domain (cyan). The close-up panel illustrates the position of the compound inside the active site. (**b**) Detailed view of the binding site. The Zn^2+^ ion is shown as a green sphere. Residues either coordinating Zn^2+^ or interacting with 3-isopropenyl-tropolone are depicted in orange. The sigma-A weighted 2Fo – Fc electron density map is shown as a green mesh (σ = 2). The sigma-A weighted Fo – Fc electron density omit map has very similar features (not shown). (**c**) Chemical environment around 3-isopropenyl-tropolone. The panel was prepared with LIGPLOT^36^. (**d**) Overlay of unliganded CapF (PDB 3ST7, light gray) and CapFΔ57-70 in complex with 3-isopropenyl-tropolone. The arrow illustrates a shift of the position of Zn^2+^ ion upon binding of the compound (~1.4 Å). The coordination bond between His290 and Zn^2+^ is lost in the presence of inhibitor.

The crystal structure of the complex of CapF with inhibitor was determined at 2.4 Å resolution (Fig. 4, Table 2). The asymmetric unit contained a dimer of CapF bound to two molecules of inhibitor (*i.e*. the stoichiometry was 1:1). The value of rmsd between the coordinates of unliganded WT CapF (PDB entry code 3ST7)^12^ and CapFΔ57-70 in complex with 3-isopropenyl-tropolone is small (all-atoms rmds = 0.7 Å) demonstrating the enzyme does not undergo major conformational changes upon ligand binding. The molecule of 3-isopropenyl-tropolone is bound deep inside the active site of the cupin domain of CapF near the Zn^2+^ ion (Fig. 4a). The carbonyl and hydroxyl groups of the tropolone ring are found within the coordination sphere of Zn^2+^ at distances of 2.2 Å and 1.9 Å, respectively. The compound buries 230 ± 1 Å^2^ in the binding pocket of the enzyme, i.e. ~75% of the compound’s total accessible surface area^21^. The seven-membered ring of 3-isopropenyl-tropolone makes extensive van der Waals contacts with residues Phe262, Lys285, Gly286, His288, Phe297, Ile339 and Met351 of the cupin domain (Fig. 4b,c). No contacts with residues of the SDR domain are observed. The value of the shape complementarity factor (*Sc*) between th inhibitor and CapF was 0.74 ± 0.01, indicating greater complementarity than that between a typical antibody-antigen complex (0.64–0.68)^22^.

Superposition of the coordinates of bound and unbound CapF revealed a shift in the position of Zn^2+^ by approximately 1.4 Å towards the tropolone ligand, resulting in the disruption of the coordination bond between Zn^2+^ and the residue His290 (Fig. 4d). The disruption of the coordination sphere of Zn^2+^ by the inhibitor results in a significant decrease in the thermal stability of CapF in comparison with the apo protein (*ΔT*_*M*_ = 5.2 ± 0.4 °C for WT CapF and *ΔT*_*M*_ = 6.3 ± 0.2 °C for CapFΔ57–70) (Table 1, Supplementary Information Figure S6). Taken together, the interaction between 3-isopropenyl-tropolone and CapF seems specific because of the combination of strong coordination bonds to the metal and the high shape complementarity with residues of the binding pocket of the cupin domain.

### Structure-activity relationship

**Figure 5:**
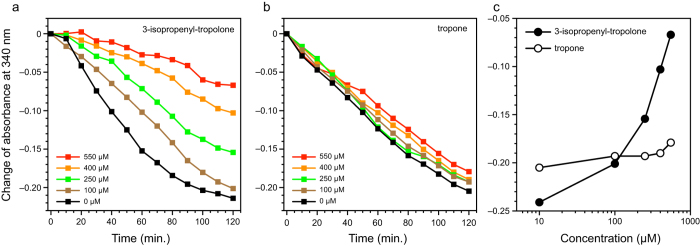
Consumption of NADPH by CapF in the presence of compounds. (**a**) Data correspond to 3-isopropenyl-tropolone. (**b**) Data correspond to tropone. (**c**) The change of absorbance after 2-hours is compared between each compound. Higher values of absorbance indicate lower consumption of NADPH, i.e. stronger inhibitory effect.

We examined several analogs of 3-isopropenyl-tropolone, namely tropolone, 4-isopropyl-tropolone, tropone, and 2-Cl-tropone, to rationalize the basis of their interaction with CapF (Supplementary Information Figure S7). To evaluate the relative inhibitory potency of the compounds we measured the relative consumption of NADPH. The results indicate that the three derivatives of tropolone (3-isopropylen-tropolone, tropolone, and 4-isopropyl-tropolone) inhibited the consumption of NADPH in a concentration-dependent basis (Fig. 5, Supplementary Information Figure S7). In contrast, the two analogs of tropone (tropone and 2-Cl-tropone) lacking the crucial hydroxyl group chelating the Zn^2+^ ion did not significantly inhibit the enzymatic activity of CapF.

ITC was employed to investigate the dissociation constant and thermodynamic signature of binding. Consistent with the inhibition experiments from above, binding of tropone and 2-Cl-tropone to CapF was not detected, whereas 3-isopropylen-tropolone and tropolone bound to CapF with similar affinity (27 ± 12 and 38 ± 7 μM, respectively) and comparable thermodynamic signatures (Fig. 3a, Table 3, and Supplementary Information Figure S8). In particular, the thermodynamic data indicated that the binding of these two tropolone compounds is driven by the enthalpy term (*ΔH* < 0), suggesting favorable non-covalent interactions between the compounds and the protein. We note that the change of entropy was also favorable, and of similar magnitude to the value of *ΔH* in these two tropolone compounds (Table 3). In contrast, the dissociation constant of 4-isopropyl-tropolone was substantially larger (*K*_*D*_ = 1.8 mM) indicating weaker affinity and resulting in very little exothermicity. Because of the low exothermic heat recorded during the titration with 4-isopropyl-tropolone, the value of enthalpy was not reliable and hence is not reported. Overall, these data demonstrate the essential role of the hydroxyl group in position C2 of the ring for metal chelation, and the unfavorable effect of enlarging the tropolone moiety at position C4 of the ring, but not at position C3, for the binding affinity.

## Discussion

The data presented in this work have revealed that natural tropolones are robust inhibitors of CapF, and well-suited for optimization to increase their potency as we explain below. First, these compounds are derivatives of the natural compound tropone, belonging to a family of compounds with diverse biological activities^23^. A recent report has suggested that tropolones are potent inhibitors of various metalloenzymes showing high ligand efficiencies (*LE* = −*ΔG*/*HAC*, where *ΔG* is the binding free energy, and *HAC* is the number of heavy atoms)^24^. In particular, the high value of *LE* observed for 3-isopropenyl-tropolone (*LE* = 0.52 kcal mol^−1^) and tropolone (*LE* = 0.67 kcalmol^−1^) found with CapF suggest that this family of compounds has the potential of being potent inhibitors of CapF^24^. Second, based on the structure-activity relationship analysis carried out and the crystallographic data obtained, these compounds seem amenable to optimization (to increase affinity and specificity) using rational structural-based ligand design methodologies, such as the target-based drug discovery and the lead optimization methodologies for the design of antibiotic agents^25^,^26^. And third, the thermodynamic data demonstrate that the binding of tropolones to CapF is driven by favorable changes of enthalpy. It is generally considered that among compounds of fragment-size, exothermic binding is an important advantage for ligand optimization because enthalpic binding accomplishes highly oriented interactions making these candidates less promiscuous binders^27^,^28^.

In summary, we performed a fragment-based screening to find suitable inhibitors of the antibacterial target CapF, a bifunctional enzyme of the biosynthetic route of CP of *S. aureus*. We identified the hit compound 3-isopropenyl-tropolone inhibiting the first reaction of CapF. Calorimetry and X-ray crystallography demonstrates that 3-isopropenyl-tropolone binds to CapF exothermically, forming coordination bonds with the crucial Zn^2+^ ion and extensive non-covalent interactions with residues of the active site of the cupin domain. We expect that the extension of the ring at the C3-position of the tropolone ring will yield compounds of increasing potency. We propose tropolones as plausible candidates to inhibit the synthesis of the capsular polysaccharide of pathogenic strains of *Staphylococcus aureus*.

## Methods

### Materials

The fragment library employed in this study was provided by the Drug Discovery Initiative (The University of Tokyo, Japan). This library, comprising 1,994 compounds, was designed to contain commercially available compounds excluding those with potential reactive chemical structures, but securing diversity from chemical descriptors. The library is compliant with the “rule of three”. Compounds are soluble to at least 200 μM in standard buffers at neutral pH when supplemented with 2% DMSO. The analogs of tropone and tropolone were purchased from commercial suppliers.

### Protein expression and purification

CapF was expressed and purified as described previously^12^. Briefly, CapF with a His_6_-tag at the N-terminal was expressed in *Escherichia coli* Rosetta2 (DE3) cells. Cells were harvested and disrupted by sonication in lysis buffer containing 50 mM TRIS-HCl (pH 8.0) and 500 mM NaCl. The cell lysate was centrifuged at 40,000 *g* for 30 min at 4 °C, and the supernatant loaded on a HisTrap column (GE Healthcare) equilibrated with lysis buffer. Protein was eluted with buffer supplemented with 0.5 M imidazole. Protein fractions containing CapF were pooled together, concentrated and loaded on a HiLoad 26/60 Superdex 200 gel-filtration chromatography column (GE Healthcare) equilibrated with lysis buffer. Fractions of CapF were concentrated and stored as above. CapF without additional treatment was employed for the screening step, NADPH consumption assay, binding concentration dependence assay by surface plasmon resonance (SPR), and inhibition assay by HPLC. When metal-depleted CapF was required, the purified enzyme was dialyzed with HEPES buffered-saline (20 mM HEPES, 150 mM NaCl, pH 7.4, abbreviated HBS) containing 1 mM EDTA, followed by a second dialysis with HBS. To prepare CapF fully occupied with Zn^2+^, the enzyme was dialyzed with HBS supplemented with 1 μM ZnCl_2_, followed by a dialysis in HBS without Zn^2+^. CapF was subjected to size exclusion chromatography to remove aggregated material after the second dialysis with HBS. The Zn^2+^-reconstituted CapF was employed in calorimetric and NADPH consumption assays. Purification of the enzyme CapE (required for the enzymatic assays) was carried out as described earlier^12^.

### Screening by SPR

Fragment screening was conducted in a Biacore T200 instrument (GE Healthcare). CapF was immobilized on a CM5 sensor chip (GE Healthcare) by the amine-coupling method^29^. Sensorgrams were obtained by injecting the compounds into a sensor chip decorated with CapF in a buffer composed of 10 mM HEPES, pH 7.4, 150 mM NaCl, 0.005% (v/v) Tween-20, 5% (v/v) DMSO, and 1 mM DTT at a flow rate of 30 mL min^−1^. Positive and negative control assays were performed periodically to confirm the stability of the protein on the sensor chip by injecting NADPH (100 μM) and buffer, respectively. NADPH was chosen for the control assays because it exhibits a suitable affinity for CapF (*K*_*D*_ = 1.6 μM)^12^. Measurements were carried out at 25 °C.

### Inhibition assay

The enzymatic performance of CapF was evaluated by examining the consumption of NADPH spectrophotometrically, or by monitoring the disappearance of substrate and the formation of product by HPLC^9^,^12^. First, the time-dependent consumption of NADPH (monitored from a decrease of adsorption at 340 nm) was carried out in 384-well plates at a final volume of 50 μL. The solution contained 1.0 μM CapE, 0.02 μM CapF, 125 μM UDP-D-GlcNAc (substrate), 250 μM NADPH and fragment compounds at various concentrations in HBS supplemented with 0.005% (v/v) Tween-20 and 5% (v/v) DMSO. Each reaction mixture was incubated at 37 °C and the absorption at 340 nm was measured with a plate reader (PHERAstar, BMG Labtech, Durham, NC).

For the HPLC-based inhibition assay, a solution containing 1.0 μM CapE, 0.02 μM CapF, 250 μM UDP-D-GlcNAc, 250 μM NADPH, and 1 mM compound in HBS buffer supplemented with 0.005% (v/v) Tween-20 and 5% (v/v) DMSO were incubated at 37 °C for 2 hours. The reaction was stopped by addition of 100 μL of ice-cold phenol/chloroform/isoamyl alcohol in a 25:24:1 molar ratio. The supernatant containing the products of the reaction was mixed with 100 μL of chloroform and examined by HPLC using a CarboPac PA1 anion-exchange column (Dionex) as described previously^9^,^12^. Identification of monosaccharides in the elution profile was based on a previous report^9^.

### Isothermal titration calorimetry (ITC)

Binding constants and thermodynamic parameters of the interaction between CapF and fragment compounds were determined with an iTC200 instrument (GE Healthcare) at 25 °C. Samples were equilibrated in a buffer composed of 10 mM HEPES (pH 7.4) and 150 mM NaCl supplemented with 5% (v/v) DMSO. CapF at 150–270 μM was titrated with compounds at 0.5–1.65 mM. Analysis of the data was performed with ORIGIN7 using a one-site binding model.

### Differential scanning calorimetry (DSC)

Thermal stability of CapF was examined by DSC in a ultrasensitive VP-capillary micro-calorimeter (Microcal). Prior to the experiment the protein was equilibrated in a buffer composed of 50 mM HEPES (pH 7.5), 50 mM NaCl and 5% DMSO. The concentration of protein and inhibitor were adjusted to 24 μM and 240 μM, respectively. The samples were heated between 10 and 110 °C at a scan rate of 1 °C/min. Data analysis was carried out with ORIGIN7 using a two-state transition model.

### Fluorescence measurement

Fluorescence measurements were carried out using the Zn^2+^-sensitive dye FluoZin-3 (Life Technologies) in a buffer composed of 10 mM HEPES, 150 mM NaCl (pH 7.4) supplemented with 5% (v/v) DMSO at 25 °C. The fluorescence intensities were measured in a plate reader (PHERAstar, BMG Labtech, Durham, NC) with excitation and emission wavelengths set to 490 nm and 520 nm, respectively.

### Expression, purification, and crystallization of CapFΔ57-70

The sequence of CapFΔ57–70 was inserted in a modified pET26 vector with a His_6_-SUMO-GGS tag at the N-terminus. The protein was expressed in *Escherichia coli* BL21(DE3) cells and induced with 0.5 mM isopropyl β-D-1-thiogalactopyranoside at 20 °C for 12 hours. Cells were harvested, resuspended in lysis buffer supplemented with 20 mM imidazole, sonicated, and the cell debris discarded after a centrifugation step at 40,000 × g for 30 min at 4 °C. The supernatant was applied onto a Ni-NTA agarose column (Qiagen) and the protein eluted with a solution containing 300 mM imidazole. The protein fractions were treated with the protease Ulp1 at 1% (w:w) and simultaneously dialyzed in a buffer containing 20 mM Tris-HCl, 50 mM NaCl, 1 mM DTT, and 10 μM ZnCl_2_ at 4 °C overnight. After dialysis the samples were passed again through a Ni-NTA column. The flow-through sample was concentrated and subjected to size exclusion chromatography in a HiLoad 26/60 Superdex 200 pg column (GE Healthcare) in a buffer composed of 50 mM HEPES and 50 mM NaCl (pH 7.5). The fractions containing truncated CapF were concentrated to 10 mg ml^−1^ and used for crystallization trials. After one round of screening using an Orix8 instrument (Douglas Instruments), single crystals were obtained by mixing 2–3 μL of protein with 2–3 μL of reservoir solution containing 200 mM KH_2_PO_4_, 10% PEG 3,350, and sodium cacodylate (pH 7.2). Suitable crystals of CapF was transfer to a new drop containing 1.8 mM 3-isopropyl-tropolone dissolved in 5% DMSO for three hours, after which they were briefly transferred to the same solution supplemented with 20% glycerol, and stored in a vessel containing liquid N_2_ until data collection.

### Data collection and refinement

Diffraction data were collected at beamline BL5A of the Photon Factory (Tsukuba, Japan) under cryogenic conditions (100 K). Diffraction images were processed with the program MOSFLM and merged and scaled with the program SCALA of the CCP4 suite^30^. The three dimensional structure of truncated CapF was determined by the method of molecular replacement using the coordinates of the full-length protein (PDB entry code 3ST7)^12^ with PHASER^31^. The initial model was refined with REFMAC5^32^ and COOT^33^. The coordinates of 3-isopropenul-tropolone were generated with PRODRG^34^. Validation was carried out with PROCHECK^35^.

## Additional Information

**Accession codes:** The coordinates and structure factors of CapF in complex with 3-isopropenyl-tropolone have been deposited in the PDB under accession code 4YRD. 

**How to cite this article**: Nakano, K. *et al*. Discovery and characterization of natural tropolones as inhibitors of the antibacterial target CapF from *Staphylococcus aureus*. *Sci. Rep*. **5**, 15337; doi: 10.1038/srep15337 (2015).

## Supplementary Material

Supplementary Information

## Figures and Tables

**Figure 1:**
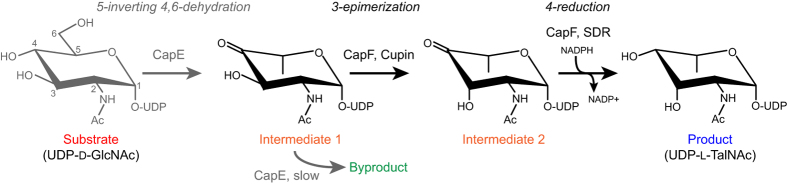
Synthesis of the CP precursor UDP-L-TalNAc by enzymes CapE/CapF. In the absence of CapF, the enzyme CapE converts the intermediate 1 to the thermodynamically favored byproduct^9^.

**Table 1:** Thermal stability of CapF.

Sample	*T*_*M*_ (°C)	Δ*H*_*DSC*_ (kcal/mol)
WT CapF	59.9 ± 0.2	176 ± 2
WT CapF + 3-isopropenyl-tropolone	54.7 ± 0.2	120 ± 1
CapFΔ57-70	60.7 ± 0.1	97 ± 2
CapFΔ57-70 + 3-isopropenyl-tropolone	54.4 ± 0.1	96 ± 1

**Table 2:** Data collection and refinement statistics.

Data Collection	CapF + 3-isopropenyl-tropolone
Space Group	C 2 2 2_1_
Unit cell	
*a, b, c* (Å)	78.58, 194.62, 158.36
α, β, γ (°)	90.0, 90.0, 90.0
Resolution (Å)	53–2.44 (2.57–2.44)
Wavelength	1.000
Observations	256,846 (34,378)
Unique reflections	42,336 (5,788)
*R*_*merge*._ (%)^a^	12.9 (57.6)
*R*_*p.i.m.*._ (%)^b^	5.5 (24.9)
*I*/*σ (I)*	8.8 (2.8)
Multiplicity	6.1 (5.9)
Completeness (%)	93.4 (89.0)
Refinement	
Resolution (Å)	53–2.44
Reflections work/free	40,164/2,139
*R*_*work*_/*R*_*free*_ (%)^c^	22.9/26.1
No. protein chains	2
No. residues	687
No. protein atoms	5,566
No. Zn^2+^ ions	2
No. inhibitor molecules	2
No. waters molecules	197
Protein B-factor (Å^2^)	31.8
Zn^2+^ B-factor (Å^2^)	36.1
Inhibitor B-factor (Å^2^)	37.3
Water B-factor (Å^2^)	21.9
Ramachandran Plot	
Preferred Regions (%)	92.8
Allowed Regions (%)	7.2
Outliers (%)	0
RMSD Bond (Å)	0.013
RMSD Angle (°)	1.6
PDB identification code	4YRD

Statistical values given in parenthesis refer to the highest resolution bin. Values were taken from 5% of data not included in the refinement.

^a^*R_merge_ = Σ_hkl_ Σ_i_ |(I(hkl)_i_ − [I(hkl)]|/Σ_hkl_ Σ_i_ I(hkl).*

^b^*R_p.i.m._ = Σ_hkl_ (I/(n_hkl_ − 1))^1/2^ Σ_i_|I(hkl)_i_ − [I(hkl)]|/Σ_hkl_ Σ_i_ I(hkl).*

^c^*R_work_ = Σ_hkl_ |F(hkl)_o_ − [F(hkl)_c_]|/Σ_hkl_ F(hkl)_o_; R_free_* was calculated as *R_work_,* where F(hkl)_o_ values were taken from 5% of data not included in the refinement.

**Table 3:** Binding parameters of analogs of tropone to CapF.

Compound	*K*_*D*_*(μM)*	Δ*H°* (kcal/mol)	*−T*Δ*S°* (kcal/mol)	*n*
3-isopropenyl-tropolone	27 ± 12	*−*3.4 ± 0.9	*−*2.8 ± 1.1	1.2 ± 0.1
tropolone	38 ± 7	*−*2.9 ± 0.8	*−*3.2 ± 1.0	1.1 ± 0.2
4-isopropyl-tropolone^a^	1,800	n.d.^a^	n.d.^a^	1.0
tropone	n.d.^b^	n.d.^b^	n.d.^b^	n.d.^b^
2-Cl-tropone	n.d.^b^	n.d.^b^	n.d.^b^	n.d.^b^

^a^The binding stoichiometry was fixed to n = 1.

^b^could not determined.
