# Supplementary material for: Discovery and characterization of natural tropolones as inhibitors of the antibacterial target CapF from Staphylococcus aureus
Source: Sci Rep. 2015 Oct 16;5:15337. doi: 10.1038/srep15337 (PMC5393024; doi:10.1038/srep15337)
Supplement: Supplementary Information [file srep15337-s1.pdf]

Supplementary Information

DISCOVERY AND CHARACTERIZATION OF NATURAL TROPOLONES AS INHIBITORS OF THE ANTIBACTERIAL TARGET CAPF FROM *STAPHYLOCOCCUS AUREUS*

Koichiro Nakano,[1,‡] Takeru Chigira,[2,‡] Takamitsu Miyafusa,[3,4] Satoru Nagatoishi,[5] Jose M. M. Caaveiro[,5] and Kouhei Tsumoto[*,1,2,3,5]*

[1]*Department of Medical Genome Sciences, Graduate School of Frontier Sciences, The University of Tokyo, 4-6-1 Shirokanedai, Minato-ku, Tokyo 108-8639, Japan,* [2]*Department of Chemistry and Biotechnology, School of Engineering, The University of Tokyo, Bunkyo-ku, Tokyo 113-8656, Japan,* [3]*Institute of Medical Science, The University of Tokyo, 4-6-1, Shirokanedai, Minato-ku, Tokyo 108-8639, Japan,* [4]*Biomedical Research Institute, National Institute of Advanced Industrial Science and Technology, Central 6, 1-1-1 Higashi, Tsukuba, Ibaraki 305-8566, Japan, and* [5]*Department of Bioengineering, School of Engineering, The University of Tokyo, 7-3-1 Hongo, Bunkyo-ku, Tokyo 113-8656, Japan*

‡These authors contributed equally to this work.

*E-mail, jose@bioeng.t.u-tokyo.ac.jp; Phone, +81-3-6409-2129.

*E-mail, tsumoto@bioeng.t.u-tokyo.ac.jp. Phone, +81-3-5449-5316.

Page S2: Fragment screen with SPR (Figure S1).

Page S3: Inhibitor has no measurable effect on the activity of CapE (Figure S2).

Page S4: Zn^{2+} governs binding of 3-isopropenyl-tropolone to CapF (Figure S3).

Page S5: Binding of 3-isopropenyl-tropolone to CapF lacking His_6-tag (Figure S4).

Page S6: Crystal contact between dimers of CapF mediated by the loop 57-70 (Figure S5).

Page S7: Thermal stability of CapF (Figure S6).

Page S8: Consumption of NADPH (Figure S7).

Page S9: Binding of analogs determined by ITC (Figure S8)

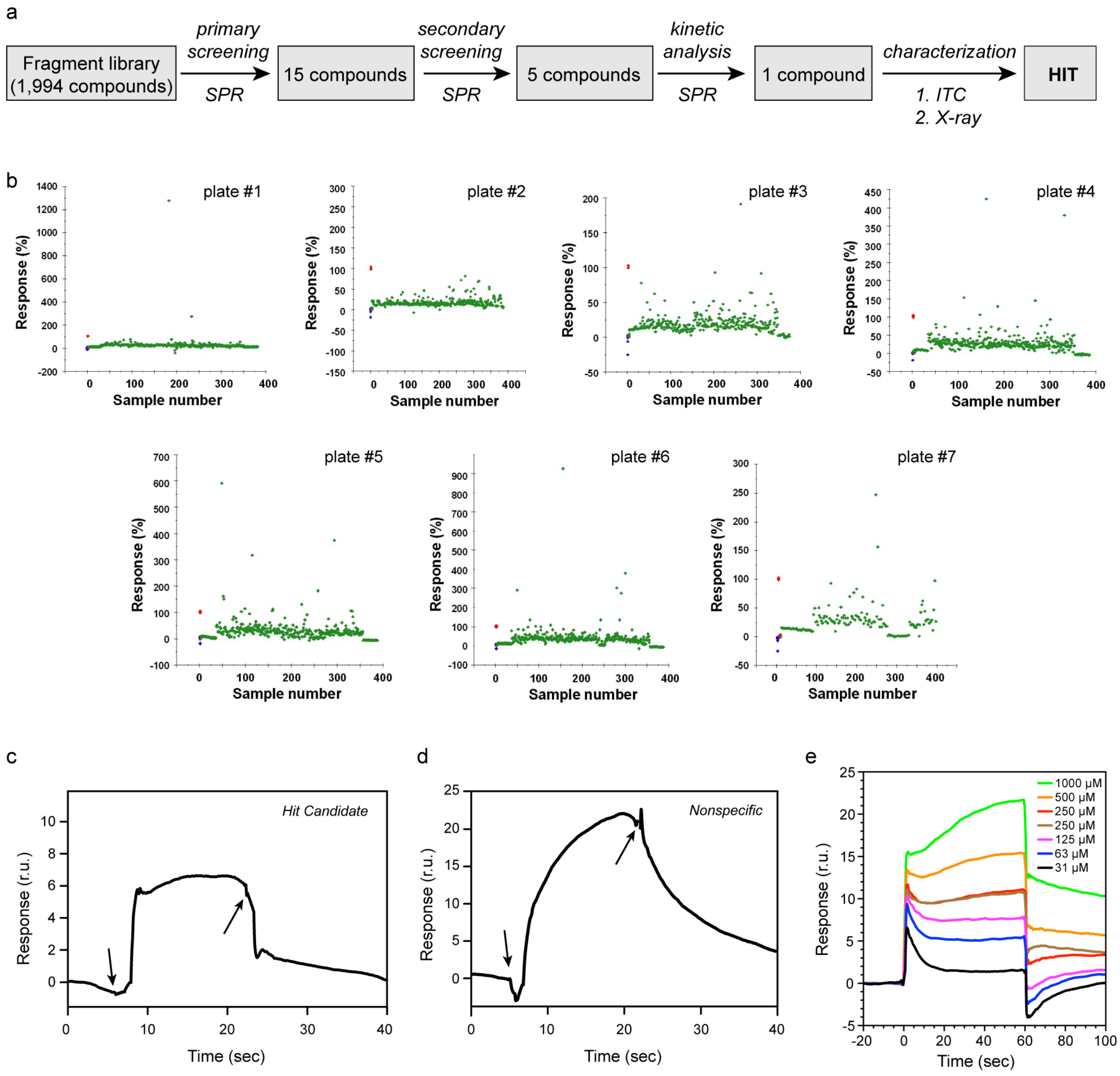

Figure S1. (a) Strategy to identify inhibitors of CapF. (b) Binding responses of fragments in the screening stage. Red, blue and green circles represent the response level after injection of NADPH (positive control), buffer (negative control), and fragment compounds, respectively. (c) Representative sensorgrams of a hit candidate. The arrows pointing upwards and downwards correspond to the injection of compound and buffer, respectively. (d) Representative sensorgram of a non-specific (or promiscuous) compound. (e) SPR binding response of 3-isopropenyl-troplone to a surface decorated with CapF. Non-specific binding is observed at the highest concentration tested (green sensorgram). Large spikes appearing just after injection of compounds or buffer were removed from panels c-e for clarity purposes.

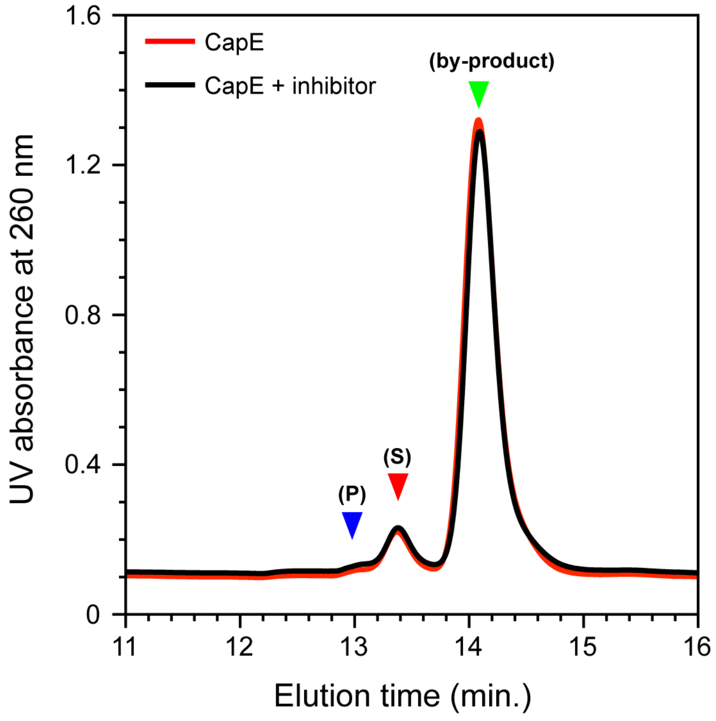

Figure S2. Elution profile of compounds after incubation of the substrate UDP-D-GlcNAc with CapE in the absence (red line) and presence (black line) of 3-isopropenyl-tropolone. In this experiment CapF was not present. Experimental conditions are described in the Methods section. The blue, red and green triangles indicate the elution peaks of product, substrate, and by-product, respectively.

(a) (b)

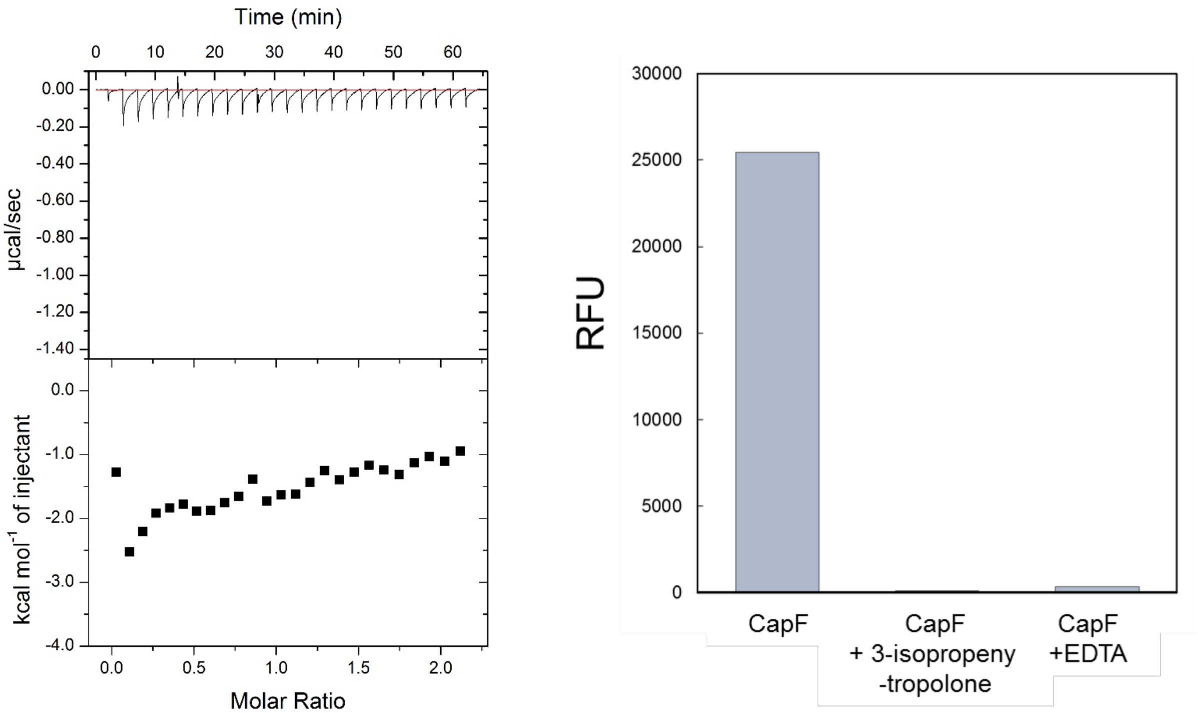

Figure S3. Zn^{2+} governs the binding of inhibitor to CapF. (a) Titration of 150 μM Zn^{2+} (prepared from $ZnCl_2$) with 1.65 mM 3-isopropenyl-tropolone. (b) Fluorescence of the Zn^{2+}-sensitive dye FluoZin-3 (1.0 μM) in the presence of CapF (1 μM), CapF (1 μM) with 3-isopropenyl-tropolone (1.0 mM), or apo-CapF.

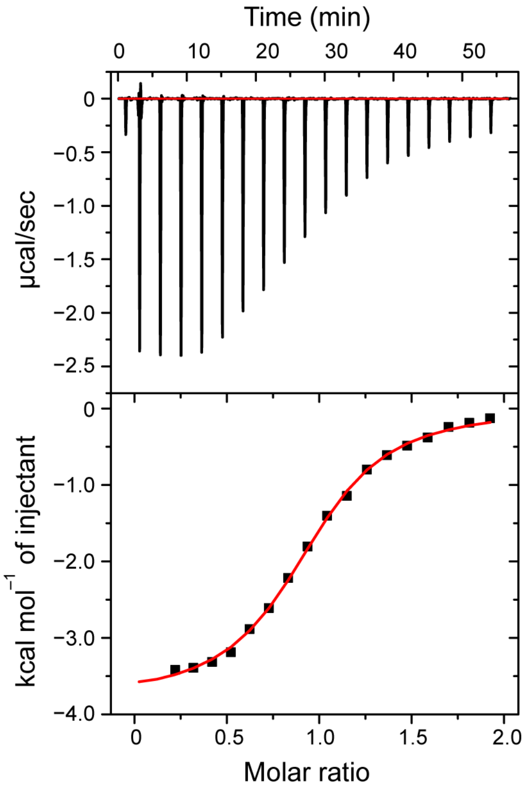

Figure S4. Binding of 3-isopropyl-tropolone to CapF lacking the His_6-tag. Titration of CapF (220 µM) with 2.2 mM inhibitor in a buffer composed of 10 mM HEPES (pH 7.5), 150 mM NaCl, and 5% DMSO at 25 °C. A construct of full-length CapF bearing a cleavable His_6-tag was prepared and purified exactly as that of CapF Δ57-70 (see Methods section for the detailed protocol of CapFΔ57-70), except that (i) the dialysis buffer after to reconstitute the enzyme with Zn^{2+} contained 150 mM NaCl (instead of 50 mM) and 12.5 µM $ZnCl_2$ (instead of 10 µM), and (ii) the buffer of the size exclusion chromatography step contained 10 mM HEPES (instead of 50 mM) and 150 mM NaCl (instead of 50 mM). The values of the thermodynamic parameters determined with ORIGIN were K_D = 13 ± 1 µM, ΔH = -3.6 ± 0.3 kcal mol^{-1}, -$T\Delta S$ = -3.0 ± 0.4 kcal mol^{-1}, and n = 0.92 ± 0.1.

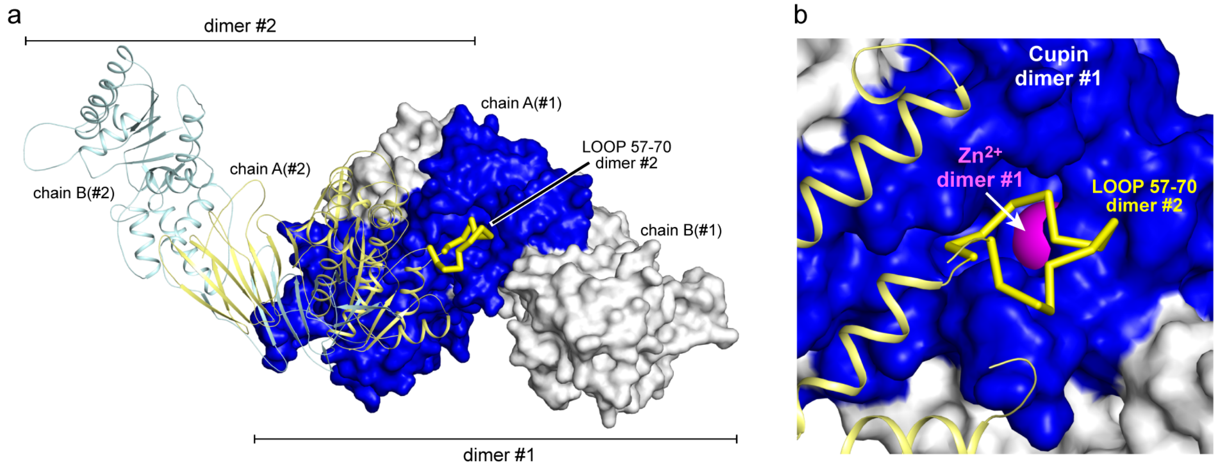

Figure S5. Crystal contact between two dimer assemblies of WT CapF (PDB entry code 3ST7) mediated by the loop 57-70. (a) Overview. The complete structure of two different dimers of CapF is shown. One dimer is shown in surface representation, and the other in cartoon representation. The loop 57-70 of chain A of dimer #2 (thick yellow ribbon) inserts in the pocket of the cupin domain of chain A of dimer #1. (b) Close up view highlighting the loop 57-70 (yellow ribbon) and the zinc ion (large magenta sphere).

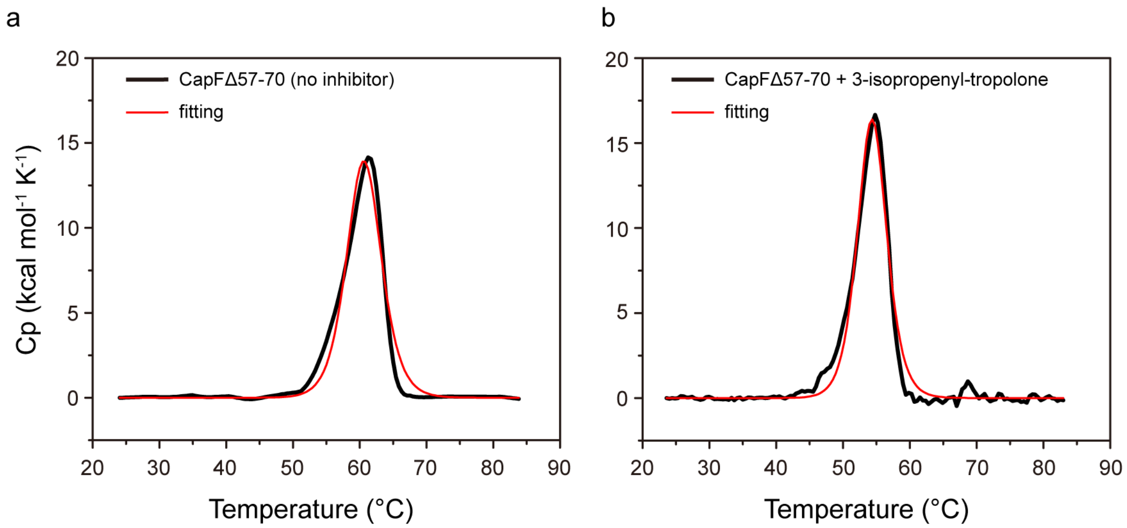

Figure S6. Thermal stability of CapF examined with DSC. (a) CapFΔ57-70, and (b) CapFΔ57-70 in the presence of 3-isopropenyl-tropolone. Concentration of CapF and inhibitor was 24 μM and 240 μM, respectively. Experimental data and fitting curves are represented in black and red lines, respectively. Thermodynamic parameters are given in Table 2.

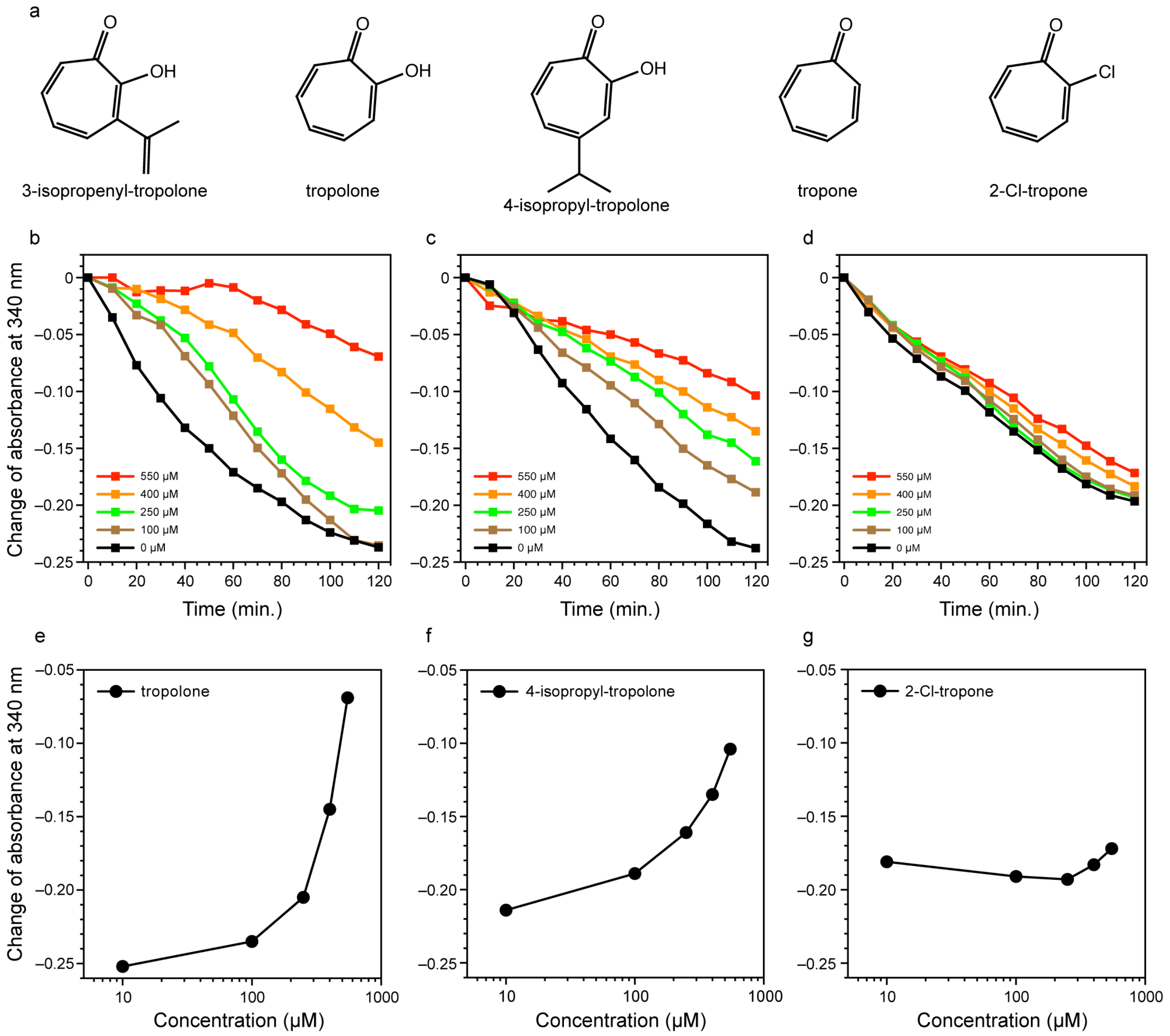

Figure S7. (a) Structure of compounds employed. (b-d) Time course of NADPH consumption by CapF in the presence of (b) tropolone, (c) 4-isopropyl-tropolone, and (d) 2-Cl-tropone. Increasing the concentration of compounds decreases the rate of consumption of NADPH. (e-g) Inhibition after two hours for (e) tropolone, (f) 4-isopropyl-tropolone, and (g) 2-Cl-tropone. Concentration of CapE and CapF was 1.0 μM and 0.02 μM, respectively. Concentration of NADPH and UDP-D-GlcNAc was 250 μM and 125 μM, respectively. The change of absorbance was calculated with respect to a control assay in the absence of CapF.

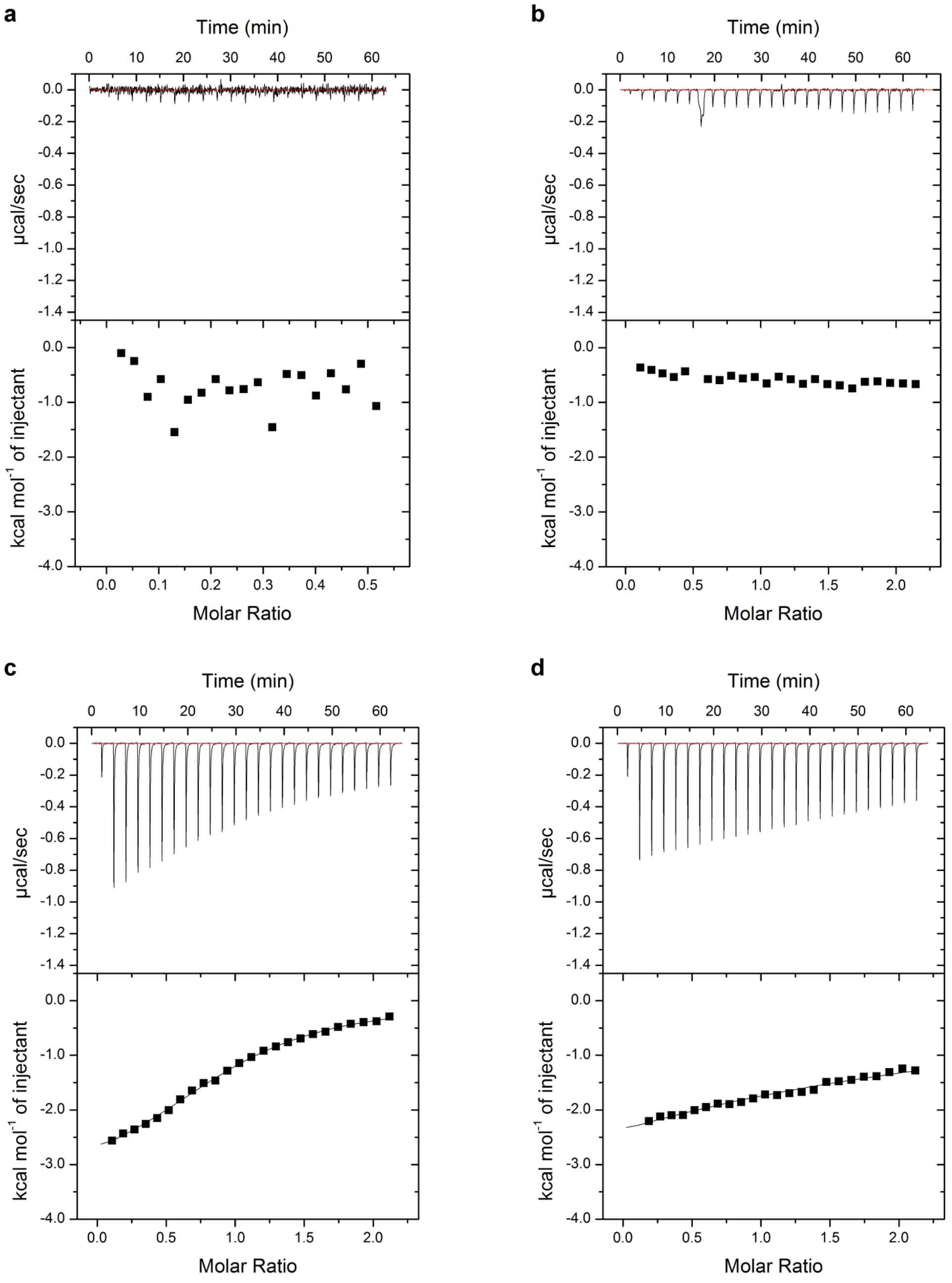

Figure S8. Binding isotherms of compounds with CapF as determined by ITC. (a) tropone, (b) 2-Cl-tropone, (c) tropolone, (d) 4-isopropyl-tropolone. Concentration of CapF in the cell was 150 µM, except in panel (a) where it was 270 µM. Concentration of compounds was 1.65 mM, except in panel (a) where it was 0.5 mM. Titrations were performed in an iTC200 instrument at 25 °C. Data analysis was carried out with ORIGIN.
